# Affective, Behavioural, and Cognitive Disorders (ABCD) of Childhood and Adolescence: Renaming and Regrouping

**DOI:** 10.1192/j.eurpsy.2025.1119

**Published:** 2025-08-26

**Authors:** S. Das, S. Ghosh, S. S. Bhandari, M. N. Tripathi

**Affiliations:** 1 Psychiatry, Dhubri Medical College & Hospital, Dhubri; 2 Psychiatry, Assam Medical College & Hospital, Dibrugarh; 3 Psychiatry, Sikkim Manipal Institute of Medical Sciences, Sikkim Manipal University, Gangtok; 4 Psychiatry, Tripathi Clinic, Varanasi, India

## Abstract

**Introduction:**

Psychiatry focuses on disorders of affect, behavior, and cognition (ABC), with half of all psychiatric disorders emerging by age 14 and three-quarters by age 24. Disorders are categorized into four types: Type 1 (affective disorders like depression and anxiety), Type 2 (behavioral disorders like ADHD and ODD), Type 3 (developmental disorders like mental retardation and speech/language issues), and Type 4 (dysfunctional disorders, including psychotic illnesses and mood disorders). These categories—emotional, behavioral, cognitive, and dysfunctional—form the ABCD framework in child psychiatry.

**Objectives:**

The study aimed to categorize childhood and adolescent mental and behavioral disorders into four groups: developmental, disruptive, emotional, and dysfunctional. It also sought to examine intra- and inter-group comorbidities, and analyze the clinical variables of these groups and their comorbidities by age and sex.

**Methods:**

This was an observational cross-sectional study conducted at Gauhati Medical College Hospital (GMCH), Guwahati, Assam, from September 7, 2018, to September 6, 2019. Existing diagnostic systems do not distinguish between child/adolescent and adult mental health, and there is a lack of a unified “common language” for childhood and adolescent mental and behavioral disorders. The study aimed to address this gap, particularly in the local geo-cultural context, where data for a new classificatory system is scarce. Participants included children and adolescents admitted to the Child Psychiatry Unit or attending the Child Guidance Clinic. Demographic (age, sex) and clinical variables (ICD-10 diagnoses) were studied, with diagnoses categorized into types 1–4.

**Results:**

Total sample size was 137. Adolescents (ten to 18 years of age) were almost double in numbers to that of children (below ten years of age). Boys outnumbered girls. Adolescents were more than children in both among girls and boys (even the single third gender was an adolescent). Most of the children and adolescents were having type 3 disorders, followed by type 1 and type 2 disorders. While majority had type 3 disorders below ten years of age (children), type 1 disorders were highest in the adolescents. Type 1 and type 3 disorders were almost equally distributed among girls, while boys predominantly had type 3 disorders. Within group comorbidity was maximum with type 3 disorders. Across group comorbidity was found mostly in type 2-type 3 disorders.

**Conclusions:**

John Lennon “imagined” of having no countries and no religion too; thus, there will be nothing to kill and die for! Bono of U2 wanted to be there “where the streets have no name”! Likewise, we too dream of a land for child and adolescent psychiatry where if we cannot live without names then let us at least have a few and simple ones: ABCD!

**Disclosure of Interest:**

None Declared

